# Bringing It All Together: Resources and Next Steps

**DOI:** 10.1002/alz70858_110739

**Published:** 2025-12-26

**Authors:** Katherine Evans, Nancy Cullen

**Affiliations:** ^1^ Alzheimer's Association, Chicago, IL, USA

## Abstract

This session will present a summary of key takeaways from the session and highlight Alzheimer's Association initatives and resources involving dementia care coordiantion and GUIDE, including the Dementia Care Navigation Roundtable and the new Dementia Care Navigation Training Series.

